# The Dirty War Index: Statistical Issues, Feasibility, and Interpretation

**DOI:** 10.1371/journal.pmed.0050248

**Published:** 2008-12-16

**Authors:** Nathan Taback

## Abstract

Nathan Taback discusses statistical limitations of a new tool, the Dirty War Index, which measures rates of particularly undesirable ("dirty") outcomes inflicted on populations during armed conflict.

In this issue of *PLoS Medicine*, Madelyn Hsiao-Rei Hicks and Michael Spagat propose a new quantitative tool [1] in the emerging field of human security sciences [2]. Quantitative data about people's insecurity can lead to objective knowledge about many of the contexts of violence in the world today. Thus developing quantitative tools for use in this domain is important.

Hicks and Spagat's tool is called the Dirty War Index (DWI), which “systematically identifies rates of particularly undesirable or prohibited, i.e., “dirty,” war outcomes inflicted on populations during armed conflict” [1]. After choosing a relevant public health outcome, a DWI is calculated as the ratio of “the number of dirty cases” of an outcome to the “total number of cases.” There are many moral and legal questions regarding the division of acts of violence in armed conflict into those that are “dirty” and those—presumably—that are not “dirty.” I am not addressing these questions here. Instead, I will discuss some statistical issues that may arise in calculating a DWI, and the feasibility and interpretation of DWIs.

## Statistical Issues

Key statistical issues that may arise in the calculation and interpretation of a DWI are: (1) selection bias; (2) missing data; and (3) censoring (i.e., the value of an observation is only partially known).

### Selection bias.

Many samples that are studied in examining the health effects of conflict on civilian populations are obtained from secondary sources such as media reports or hospital records. But these secondary sources almost always use different selection criteria compared to what would be used to derive a sample in a primary study focused on health effects of conflict. The use of secondary sources inevitably leads to selection bias: the health effects of conflict upon civilians in the sample are not representative of health effects due to conflict in the civilian population as a whole.

**Linked Policy Forum**

This Perspective discusses the following new article published in *PLoS Medicine:*

Hicks MH-R, Spagat M (2008) The Dirty War Index: A public health and human rights tool for examining and monitoring armed conflict outcomes. PLoS Med 5(12): e243. doi:10.1371/journal.pmed.0050243

Madelyn Hsiao-Rei Hicks and Michael Spagat introduce the “Dirty War Index,” a public health tool that identifies rates of undesirable or prohibited war outcomes inflicted on populations during armed conflict.

If a biased sample is used to calculate a DWI, then the estimate will be inaccurate. Hicks and Spagat acknowledge that “bias can affect DWI values.” However, they argue that since a DWI is a *ratio* (rather than an absolute number), “DWIs are relatively less affected by under- or over-counting than absolute numbers.” They give the example of rape, and argue that “if a population generally under-reports war-related rape by 40%, this does not bias comparing rates between different combatant groups” [1]. This statement is true, except that it is difficult to think of an example where researchers would have an estimate of the magnitude of under-reporting especially concerning rape.

Selection bias impedes the generalizability of a DWI, but the DWI could nevertheless be sufficient for policy or legal purposes. For instance, if a biased sample of rape victims has a rape DWI of 10%, then this information might, for example, be useful in planning a prevention program or gathering evidence for a criminal investigation, even if the magnitude of the bias is not readily quantifiable.

### Missing data.

Suppose that a DWI for lethality to civilians is to be calculated, but for several civilians in the sample information is missing on their mortality status or the weapon used against them. How are these civilians counted when calculating a DWI? One solution is to use statistical techniques to deal with missing data [3] and perform sensitivity analyses to investigate the effect that the missing data has on a DWI estimate.

### Censoring.

In several types of reports of casualty deaths and injuries, some values may only be partially known, since death and injury counts are sometimes recorded in the form of, “at least *X* people were killed.” If this type of data is used to calculate a DWI then it will almost surely be an underestimate due to right-censoring (an observation is above a certain value, but it is unknown by how much). Underestimating a DWI might have adverse policy and legal implications since the problem may seem less severe.

## Feasibility

In a study that I coauthored with Robin Painter and Ben King, we used a convenience sample derived from United Nations security reports to compute a perpetrator-to-abused ratio [4]. We used this ratio to compare sexual violence between armed groups in the Democratic Republic of the Congo. The perpetrator-to-abused ratio is another quantitative tool useful in comparing patterns of sexual violence between armed groups. Calculating the rape DWI using our data would be impractical since the total number having face-to-face contact with the combatant group was unavailable in these reports. This is a specific example of a data source on the health effects of conflict on civilians where it would not be feasible to calculate the DWI suggested by Hicks and Spagat [1]. Indeed, many of the denominators used in the example of DWI calculations in Table 2 of Hicks and Spagat's paper, such as total number of civilians injured, may not be readily available for most conflicts.

## Interpretation

Interpreting certain DWIs presents a number of difficulties beyond the accuracy of the estimates. In general, low DWI values will be hard to interpret and compare. If the DWI for rape in one combatant group, say group A, is 6% and in another combatant group, say group B, is 3%, then is group B conducting a “cleaner” war than group A because their DWI for rape is one-half the rape DWI for group B? Some readers may question the value of arriving at a DWI in such contexts.

## Abbreviations

DWI: Dirty War Index
